# Spontaneous Pneumomediastinum in COVID-19 and Myasthenic-like Symptom Complications in Two Relatives: A Coincidence or Spike Toxicity with Thymic Response in Predisposed Individuals? Two Clinical Cases with a Comprehensive Literature Review

**DOI:** 10.3390/jcm15010159

**Published:** 2025-12-25

**Authors:** Barbara Brogna, Mariagrazia Nunziata, Luigi Urciuoli, Annamaria Romano, Antonietta Laporta, Claudia Brogna

**Affiliations:** 1 Unit Interventional and Emergency Radiology, St. Giuseppe Moscati Hospital, Center of National Excellence and High Speciality, 83100 Avellino, Italy; luigiurciuoli@yahoo.it (L.U.); antoniettalaporta6@gmail.com (A.L.); 2 U.O.C. Internal Medicine-Moscati Hospital, 83100 Avellino, Italy; mg.nunziata@gmail.com; 3 Unit of Pneumology, St. Giuseppe Moscati Hospital, 83100 Avellino, Italy; annareromano@gmail.com; 4 Department of Pediatric Neurology, Catholic University, 00168 Rome, Italy; claudiabrogna@yahoo.it

**Keywords:** pneumomediastinum, COVID-19, thymus hyperplasia, COVID-19 vaccination, myasthenia gravis, long COVID, spike protein

## Abstract

Pneumomediastinum (PM) in SARS-CoV-2 infections can have a multifaceted presentation. The most frequently described cases of spontaneous PM (SPM) occurred during the first waves of the SARS-CoV-2 pandemic due to alveolar fragility related to severe cases of interstitial pneumonia and vascular injury that predisposed to alveolar destruction and to the Macklin effect in PM development. Cases of SPM were also reported secondary to non-invasive mechanical ventilation (NIV) and to the increasing use of higher doses of corticosteroid therapy. However, true SPM in COVID-19 patients without any identifiable risk factors and presenting as a “Hamman syndrome” (HS) has also been observed, although it represents a very rare clinical entity. Both lung dysbiosis and spike protein toxicity could be implicated in SPM, including cases occurring after COVID-19 vaccination. Furthermore, a variety of clinical entities have been reported that are similar both in COVID-19 infection and after the related COVID-19 vaccination. We present two clinical cases (a 14-year-old boy and his mother), one presenting with SPM and both showing thymic hyperplasia, myasthenic-like symptoms, and long COVID features as a post-vaccination syndrome (PACVS). This report highlights how genetic and familial predisposition could play a role in the thymic response both in COVID-19 infection and after vaccination, involving the toxicity of the spike protein as a common denominator.

## 1. Introduction

The pandemic caused by SARS-CoV-2 shocked healthcare systems worldwide, especially when it broke out in 2020, with repercussions on different fields—from education to medical training to mental health [1].

In fact, although it shared genetic similarity with SARS-CoV-1, which was epidemic at the beginning of the 21st century in some countries, SARS-CoV-2 appeared novel due to the rapidity of its spread and the explosion of interstitial pneumonia globally [2,3,4,5]. Pneumomediastinum (PM) usually refers to the presence of air in mediastinal spaces and can also be a possible complication of acute respiratory distress syndrome (ARDS), associated with viral infections such as SARS and H1N1 interstitial pneumonia, as well as bacterial or fungal pneumonia in immunocompromised patients or those with immunological conditions [6,7,8,9,10]. The PM incidence was higher in COVID-19 patients than in the general population, especially during the first two waves of COVID-19 [11,12,13,14,15,16,17,18], when there were more frequent severe cases of pneumonia complicated by ARDS, which required intensive care unit (ICU) management with mechanical ventilation (MV). In fact, alveolar destruction associated with the double virus pathogenic mechanism—direct damage from inflammatory alveolar infiltration and indirect vascular damage associated with the development of focal ischemia and necrosis—predisposes to alveolar rupture and air migration in the lung interstitium, through the bronchovascular sheath, and into the mediastinum via the Macklin effect [18,19,20,21,22]. Similar pathological mechanisms have been previously observed in other viral pneumonias and in some connective autoimmune diseases, such as dermatomyositis [7,10,21]. In addition, the necessity for MV in patients with high blood pressure increased the probability of developing both PM and pneumothorax or other complex conditions, such as PM associated with pneumothorax [13,14,15,16,17,18,19,20,21,22,23,24,25,26,27,28].

As reported in previous studies [16,17,18,19,21,22,23,24], the development of PM in non-mechanically ventilated COVID-19 patients was a relatively uncommon complication, and it was a cause of thoracic pain in the aforementioned patients. Non-invasive PM was usually reported in COVID-19 patients with non-invasive ventilation (NIV) or oxygen supplement, and this complication showed a slightly higher prevalence during the second wave of the pandemic [16,17,18,19,21,22,23,24,25,26].

Primary spontaneous pneumomediastinum (PSPM) is usually reported in healthy subjects without obvious causative factors [28,29,30]. Young males with a low mass index are more predisposed to develop SPMs [28,29,30]. Coughing, vomiting, vigorous exercise, or Valsalva maneuvers can also cause SPMs [28,29,30]. Chest computed tomography (CT) with high-resolution reconstruction (HRCT) was the most used imaging tool during COVID-19 for evaluating the severity of pneumonia, and it has greater sensitivity for PM evaluation [20,27]. Chest CT imaging can clearly show the Macklin effect with linear collections of air near the bronchovascular interstitium and evaluate all mediastinal compartments with a prognostic and predictive role [20,27]. Nevertheless, chest CT is also used to study long-term lung changes in previously hospitalized COVID-19 patients, especially in those with moderate and severe interstitial pneumonia, as they often suffer from persistent dyspnea and impaired pulmonary function, which are frequently associated with long COVID manifestations [31,32,33,34,35,36,37]. Long COVID manifestations may also be characterized by persistent fatigue, myalgia, and chronic fatigue syndrome [36,37].

On the other hand, during the pandemic, an increased detection of mediastinal masses as thymus hyperplasia on chest CT examinations has been reported, often coincidentally [38,39]. The thymus plays an important role in regulating immune responses, as it is the main anatomical site for the production and development of T cells [38,39,40,41]. Thymic hyperplasia has been observed in COVID-19 patients, and it was usually associated with increased T lymphocyte production, which appeared to be a beneficial outcome [38,39,40,41]. However, strong immune responses such as thymic hyperplasia or swelling of the auxiliary lymph nodes have also been reported after COVID-19 vaccination [42,43,44,45,46]. Several case series have also described myasthenia gravis (MG) associated with SARS-CoV-2 infection as an early onset after viral infection or after COVID-19 vaccinations [47,48,49,50,51,52,53,54,55,56]. MG is a rare chronic neuromuscular autoimmune disease caused by antibodies against the acetylcholine receptor (AChR) or, more rarely, against muscle-specific kinase (anti-MuSK Ab) or lipoprotein-related protein 4 (LRP-4) or triple-seronegative (triple-SN) MG, in which all these antibodies are absent [52,57,58,59]. Antibodies are not detected in 10–15% of patients with generalized MG, usually because of the low sensitivity of the assay used [52,57,58,59]. The classical manifestation of classical MG is progressive muscle weakness and fatigue affecting legs, arms, neck, and face [58]. Muscle weakness typically worsens with repeated muscle activity, often presenting as mild weakness in the morning that becomes more pronounced at the end of the day [53,57,59]. Extraocular muscles are frequently affected, usually asymmetrically, with typical symptoms including ptosis and diplopia [53,57]. Most seronegative patients have a mild disorder characterized by predominant ocular manifestations [52,57,60]. Thymic dysfunction is also a well-recognized cofactor of the disease. Thymoma and thymic hyperplasia are reported in most patients with MG [52,57,61]. MG is usually subgrouped according to a type of pathogenic autoantibodies, age of onset, thymus pathology, and the degree of generalized muscle weakness [58]. However, a clinical entity more similar to long COVID symptoms after COVID-19 vaccination has recently been discovered, and it is called post-COVID-19 vaccination syndrome (PACVS), which is also characterized by MG-like symptoms [36,62,63].

## 2. Case Presentations

**Case 1**

A 14-year-old boy came into the emergency room on 16 October 2020 with fever (38 °C), cough, dyspnea, and persistent thoracic pain. He had already tested positive for SARS-CoV-2 on a nasal swab 7 days prior to hospital admission and had been on home therapy with acetaminophen for fever. The boy did not report any history of bronchial asthma, esophageal reflux, or other particular problems. During hospital admission, he exhibited normal vital parameters (peripheral oxygen saturation on room air always >96%) and laboratory values within the normal range (Table 1).

**Figure 1:**
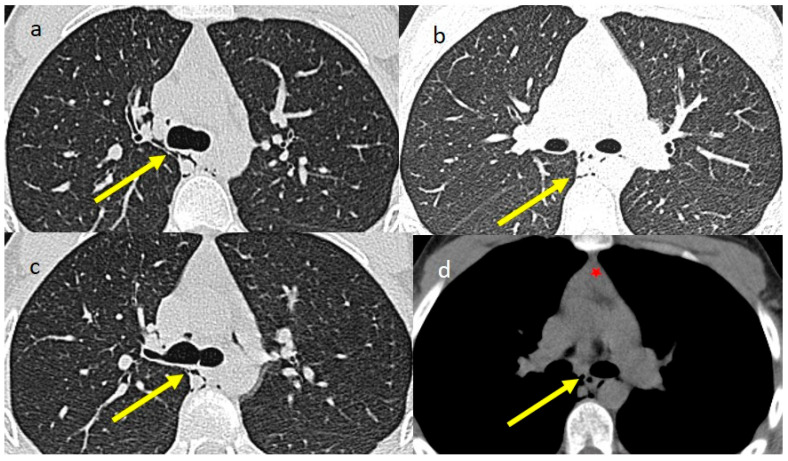
Images (**a**–**c**) show mediastinal spontaneous emphysema (shown by the yellow arrow) on the lung window of HRCT, without lung involvement; in image (**d**), the mediastinal emphysema (yellow arrow) is shown along with the residual of thymic tissue (red star) on the mediastinal window.

His BMI was 21 kg/m^2^. Both the electrocardiogram and echocardiogram results were normal. However, for persistent severe thoracic pain, chest CT was requested. The latter highlighted the presence of PM in the middle and posterior mediastinum and near the hilar region (Figure 1). No lung involvement was present, and he reported no previous episodes of vomiting, only cough. Residual thymic tissue was also visible on CT (Figure 1).

**Figure 2:**
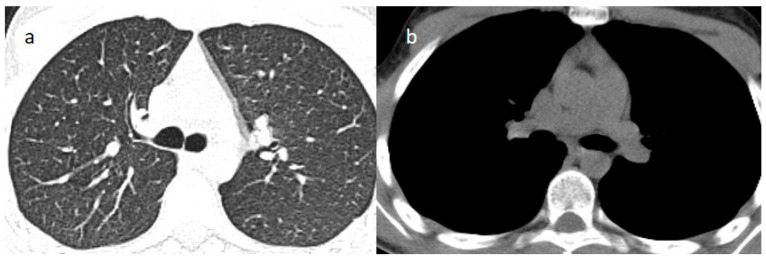
The resolution of the previous mediastinal emphysema in the lung window image (**a**) and in the mediastinal window image (**b**) on the chest CT made a week after hospital admission.

Therefore, oral corticosteroid therapy (prednisone 25 mg), protein pump inhibitor, and a prophylactic dose of low-molecular-weight heparin (LMWH) 4000 IU were started, with symptom improvement and the absence of air in the chest CT scan observed one week later (Figure 2).

**Figure 3:**
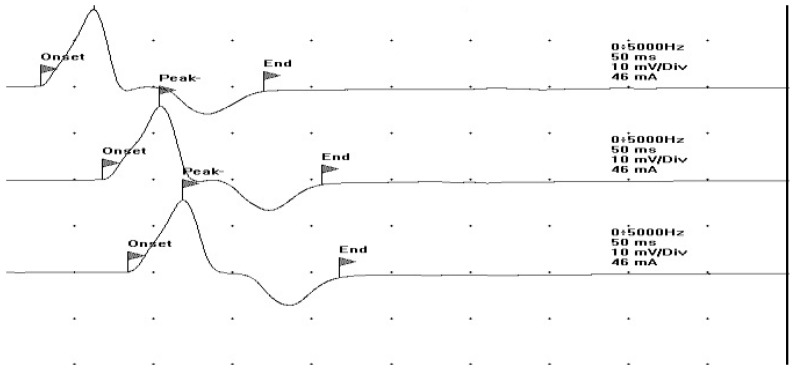
Single-fiber electromyography at the level of the ulnar nerve is within normal limits.

Esophageal injuries were also ruled out through an endoscopic study during hospitalization. Nevertheless, the boy continued to suffer from a persistent cough after COVID-19 infection for several months. In July 2021, the patient was administered the first dose of the COVID-19 mRNA vaccine BNT162b2 (Pfizer-BioNTech). About 10 days after the vaccination, he developed MG-like symptoms characterized by fatigue, muscle weakness with fatigability, and decreased proximal strength in the upper and lower limbs, resulting in an inability to perform sports, as well as convergent strabismus and upward diplopia, confirmed during a neurologic examination. The MRC (Medical Research Council) scale score was 5/5 in both upper and lower limb muscles. However, mild hypostenia at the lower limbs was evident during the Mingazzini maneuver, with a maximum holding time of 25 s. This finding mildly improved after 30 mg of pyridostigmine (Mestinon), which was administered orally, resulting in an improvement in motor deficits. Therefore, several tests were performed, including the serum AchR antibody titer with results within the range values (antibody AchR 0.01 nmol/L; normal value range: 0–0.4 nmol/L) (dosed with ELISA), and both his MuSK-Abs titer and LRP-4 were negative. Single-fiber electromyography (EMG) and repetitive nerve stimulation were within normal limits (Figure 3).

**Figure 4:**
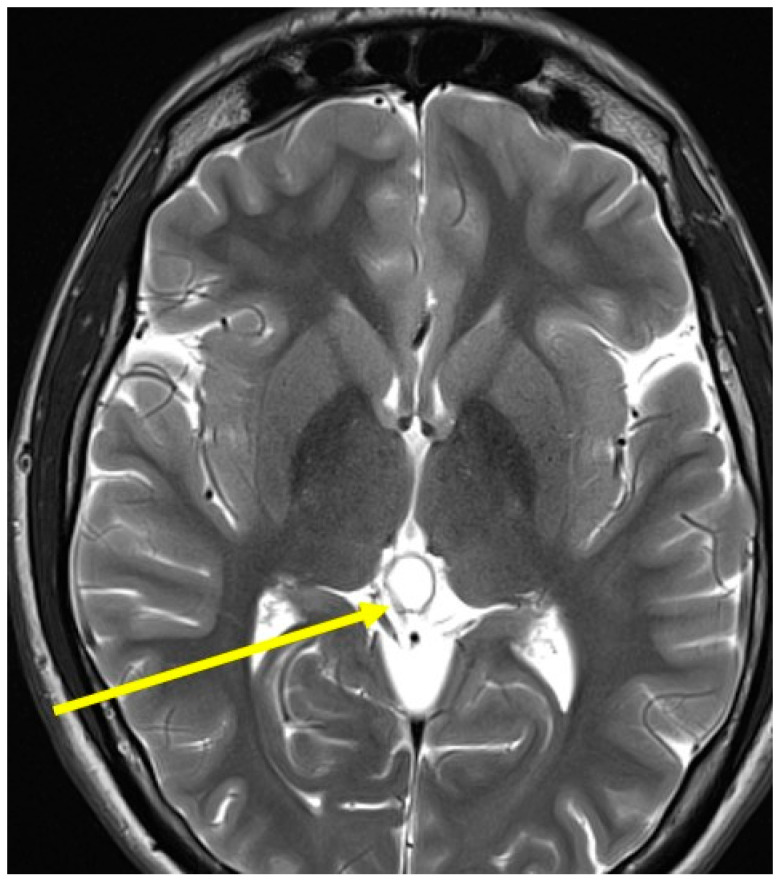
The brain MRI did not show any inflammatory lesions but only a pineal cyst (indicated by the yellow arrow).

A brain MRI resulted in negative findings for inflammatory lesions (Figure 4).

**Figure 5:**
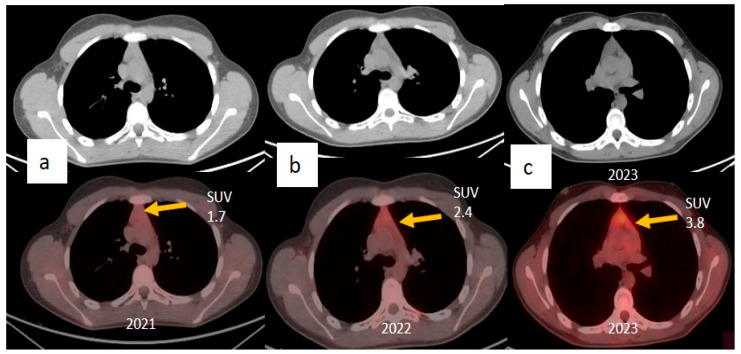
In the images, (**a**–**c**) show ^18^F-FDG PET-CT images with a progressive increase in SUV values in the thymic region from August 2021 to October 2023.

To rule out thymic pathologies, clinicians requested ^18^F-fluorodeoxyglucose-PET/computed tomography (^18^F-FDG-PET/CT), which was performed one month after vaccination, with image acquisition 60 min after radiotracer injection. The results showed a mild increase in uptake in the superior mediastinum at the thymic level (SUV 1.7). In November 2021, the boy was subsequently reinfected with SAR-CoV-2. In January 2022, he received another dose of the mRNA vaccine BNT162b2 (Pfizer-BioNTech). The FDG-PET/CT performed one month after the vaccination showed a mild increase in SUV compared with the previous examination (SUV 2.4) in the thymic region. In July 2022, he was again infected with SARS-CoV-2, and another similar infection was reported in September 2023. All these COVID-19 infections manifested with fever and cough. In October 2023, another ^18^F-FDG-PET/CT was performed, showing a further increase in SUV (SUV 3.8). All PET images are reported in Figure 5.

**Figure 6:**
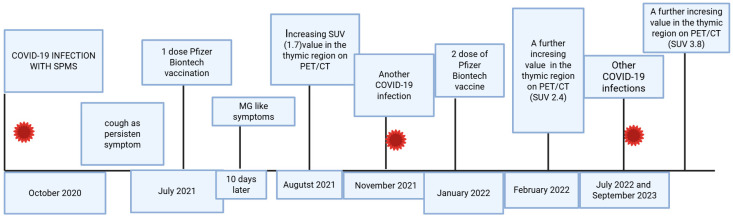
In this figure, the main events of this case are summarized: from COVID-19 infection in 2020 to vaccination and repetitive infections, with increasing values in the thymic region on PET/CT.

A thymectomy was scheduled some months later. The histology of the boy showed true thymus hyperplasia. The boy’s symptoms, such as thoracic pain and muscular weakness, showed improvement after surgery. All events are summarized in Figure 6.

**Case 2**

**Figure 7:**
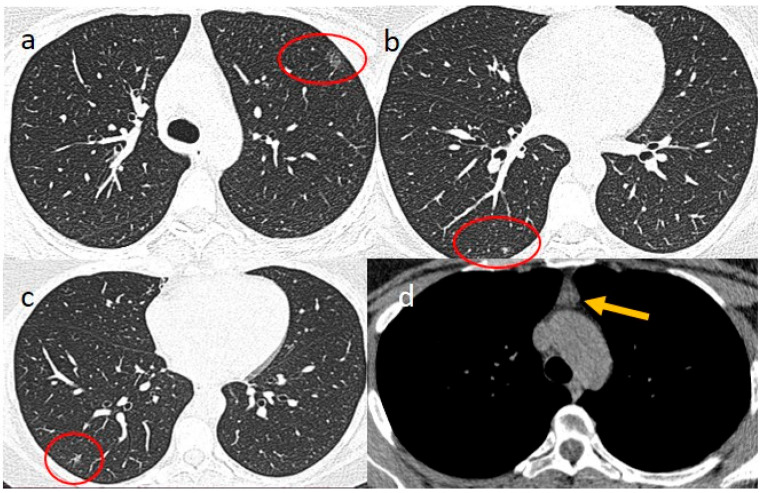
The mother’s HRCT with a few inflammatory areas (red circle) was visible in the “lung window”, as represented in images (**a**–**c**). Image (**d**) shows the thymic tissue compatible with “thymic hyperplasia” (shown by the orange arrow) in the mediastinal window.

The boy’s mother, a woman of 45 years, had also tested positive for SARS-CoV-2 on a nasal swab in the previous ten days, and she was treated with anti-inflammatory and antibiotic therapy using Zitromax. She presented diarrhoea at home, and when she arrived at the emergency department on the same day as her son, she also reported dyspnoea, cough, and thoracic pain. The mother had a history of grade IV endometriosis and had undergone a hysterectomy in 2017. Chest CT showed a few inflammatory GGOs in parenchymal areas (Figure 7), and mild thymic enlargement compatible with thymic hyperplasia was also observed (Figure 7).

However, the laboratory analysis was in the normal range (Table 2).

**Figure 8:**
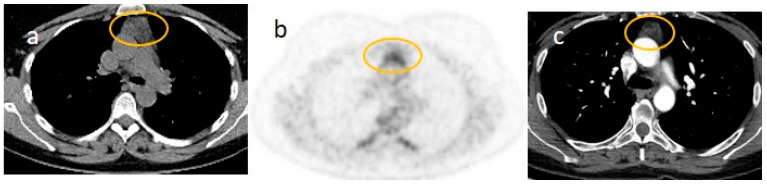
Images (**a**,**b**) show ^18^F-FDG PET/CT with a mild increase in the dimension of the previous thymic tissue (**a**) (orange circle) and a mild increase in SUV values (3.7) in (**b**) (orange circle). Image (**c**) shows the contrast CT, with poor enhancement of the thymic tissue (orange circle).

The mother had been treated with corticosteroid therapy (injectable dexamethasone at 4 mg) and LMWH 4000 IU. She had a good remission of her COVID-19 infection. Interestingly, after receiving the first dose of the Pfizer-BioNTech vaccine in May 2021, she developed asthenic symptoms, including muscle weakness, increased fatigability, and ocular ptosis, resembling an MG-like syndrome. The AChR antibody titer, in addition to MuSK-Ab and LRP-4 antibody titers, was negative. Single-fiber EMG and repetitive nerve stimulation also yielded negative results. She had another COVID-19 infection some months later (in October 2021). Another dose of mRNA vaccine (Moderna mRNA-1273) was administered at the beginning of December 2021, with fatigability and muscle weakness worsening. Therefore, ^18^F-FDG PET-CT was carried out one week later, with image acquisitions after 60 min from radiotracer injection, which showed a mild enlargement of the thymic tissue with SUV values (SUV3.7) (Figure 8). Enhanced chest CT was also carried out, showing poor contrast enhancement with respect to the thymic tissue. Due to persisting neurological signs, thymectomy was performed. On her histology, a diagnosis of true thymic hyperplasia was confirmed, and her neurological symptoms improved after the surgical intervention.

**Figure 9:**
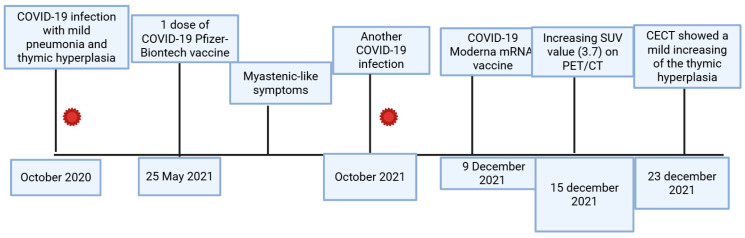
This image reports the main events from the COVID-19 infection and vaccination of the second case.

In Figure 9, the main events of this case are summarized.

## 3. Discussion

### 3.1. Secondary Non-Traumatic Pneumomediastinum Across Different COVID-19 and Spontaneous Pneumomediastinum as a Presentation of Hamman’s Syndrome

Several multifactorial symptoms have been found to be associated with an increased risk of PM in non-mechanical ventilation COVID-19 patients, such as NIV therapy, oxygen support with HFNC, the use of corticosteroid therapy, past lung diseases, and COVID-19 pathogenesis itself [15,16,17,18,19,20,21,22,23,24,25,26,27,28]. Older age and underlying lung diseases increase risks [18]. NIV therapy was commonly used during the pandemic to treat COVID-19 hypoxemia as a standalone therapy or to delay the use of MV [63]. NIV normally develops through positive end-expiratory pressure (PEEP), which may contribute to alveolar overdistension and rupture. Treatment with NIV usually includes masks, helmet continuous positive airway pressure (hCPAP), and modified snorkel masks [63,64]. The use of HFNC, although it generally delivers a much lower PEEP than NIV, can also increase the risk of PMS in COVID-19 patients, as alveoli are more prone to rupture [63]. However, PM associated with NIV or unassisted breathing has been reported during COVID-19, mainly in case reports/case series and in a few retrospective studies [7,15,16,18,19,20,21,22,23,24,25,26]. All case studies and series are summarized in Table 3.

It should be noted that cases of PM in non-mechanical ventilated COVID-19 patients have been found more frequently during the second wave of the pandemic compared to other virus waves [6,15,16,18,19,20,21,22,23,24,25,26,27]. This finding could be explained by the change in SARS-CoV-2 virulence during the pandemic [19]. In fact, the Omicron variant was associated with significantly lower clinical severity, reduced oxygen requirements, lower rates of hospitalization, and decreased mortality rates [19]. Moreover, during the first two waves of the pandemic, there were more common severe cases of interstitial pneumonia, which predisposed to more lung friability, and in the second wave, there was also an increase in the use of corticosteroid therapies for all patients requiring oxygen supplementation [6,15,16,18,20,21,22,23,24,25,26,27]. Cases of PMS in non-ventilated COVID-19 patients were usually reported for the Delta variant, with fewer cases than the second wave [19,65].

PM in non-invasive mechanically ventilated patients is a marker of poor prognosis in COVID-19 patients, with an increased rate of mortality in these patients and longer hospitalization periods [26]. A male predominance in PM development has been reported [26]. However, we also present a very rare case of SPM without any apparent causes in a young male boy who did not exhibit pneumonia involvement and who developed SPM during COVID-19 infection. In our case, the patient did not have any particular risk factors such as asthma, chronic obstructive lung disease (COPD), esophageal pathologies, or drug abuse and only presented some episodes of coughing during SARS-CoV-2 infection. On the other hand, based on Hamman’s definition, primary spontaneous pneumomediastinum (PSPM) usually occurs in healthy individuals with no demonstrable underlying diseases and traumatic events [28,29,30]. The causes of PSPM may include a history of excessive vomiting or coughing, and it occurs more commonly in young male individuals, with a benign course [28,29,30]. In our case, some reported episodes of coughing may also have been a possible cause of SPM. However, the diversity of lung microbiomes in COVID-19 patients could also be correlated with the risk of complications and associated with alveolar fragility [66]. In fact, lung dysbiosis may potentially contribute to alveolar damage via direct injury and the regulation of alveolar inflammation and immunity [66]. Furthermore, in our case, we speculate that the SPM could also be caused by SARS-CoV-2 infection on the basis of different mechanisms, including coughing [66,67,68,69]. In addition, it could also be caused by a toxicity spike during mediastinal inflammation, as it involves structures within the mediastinum, such as large blood vessels and lymphatic tissues, due to their proximity to the lungs and involvement in immune responses [66,67,68,69]. In fact, we could only find one similar case; however, it manifested after the Pfizer-BioNTech mRNA vaccine [69].

**Figure 10:**
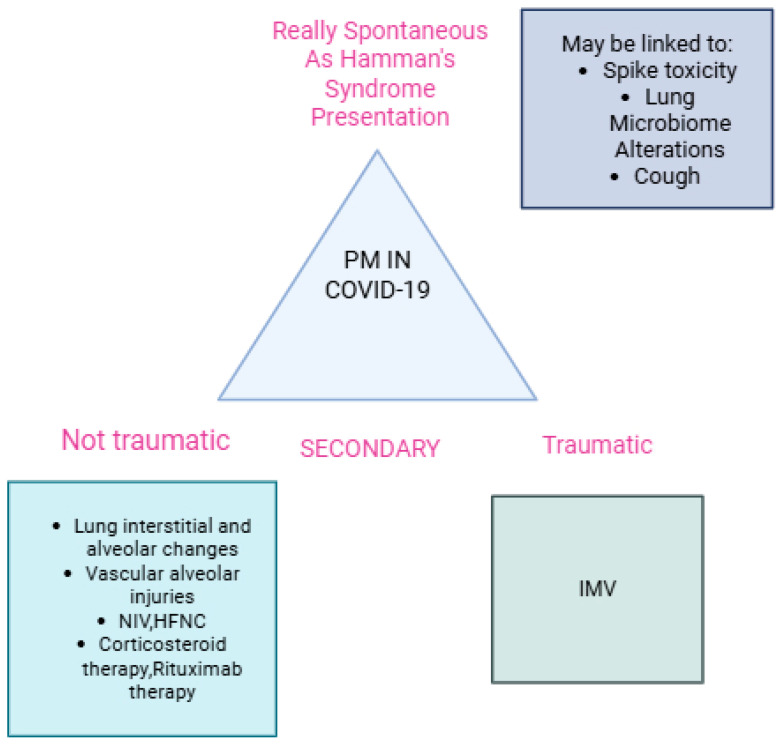
The main causes of PM in COVID-19 patients are summarized (IMV: invasive mechanical; HFNC: high-flow nasal cannula; NIV: non-invasive ventilation; PM: pneumomediastinum).

Therefore, according to Silva et al. [30], the definition of SPM should be better outlined and revised, since a PM related to an identifiable event cannot be considered truly spontaneous. Because of this consideration, the term “spontaneous pneumomediastinum” has been inappropriately applied and overused in COVID-19 patients during the pandemic. In fact, cases of PM in COVID-19 patients under NIV were reported as spontaneous. Therefore, during COVID-19, non-traumatic PM usually had multifaceted origins, ranging from lung-predisposing conditions to lung alveolar and endothelium damage caused by the virus itself through various mechanisms. However, true SPM without lung involvement was a very rare but possible complication in COVID-19 patients. We speculate that, in this case, spike toxicity and microbiome alterations can also be involved, as our patient manifested SPM without an identifiable risk factor (Figure 10).

### 3.2. Thymic Hyperplasia in COVID-19 Infection and Vaccination and the Role of Multimodality Imaging

The thymus is one of the main organs of the lymphatic system, and it usually promotes the development of T lymphocytes that protect against foreign organisms, such as bacteria and viruses [70,71]. It is located in the prevascular compartment of the mediastinum according to the International Thymic Malignancy Interest Group (ITMIG) classification, and its size varies physiologically with age, with the largest dimension in infancy and a senescence involution [72,73]. Thymic hyperplasia refers to the condition characterized by an enlargement of the thymus gland as a response to a variety of conditions, including infections, cancer, chemotherapies, and vaccines, and these cases are usually associated with true thymic hyperplasia, with an increase in normally organized thymus tissue exceeding the normal upper limit for a specific age, as determined by weight and volume [61,72,73,74]. Thymic hyperplasia can also occur in combination with some autoimmune diseases, such as MG syndrome or immunological disorders, and in these cases, it is usually associated with lymphoid hyperplasia, which is characterized by an increased number of lymphoid follicles and germinal centers in the thymus [61,71,72]. Chest X-rays have poor sensitivity in evaluating mediastinal pathologies [72]. Therefore, the diagnosis of thymus hyperplasia is usually made via chest CT and can also be detected incidentally [72,73,74]. On imaging, CT thymus hyperplasia usually appears as a diffusely enlarged thymus with a smooth, lobulated, triangular contour and homogenous enhancement [72,73,74]. On CT, it is usually possible to differentiate between lymphoid and true hyperplasia, as the latter usually shows lower attenuation values after contrast administration [75]. However, in cases of clinical uncertainty or atypical imaging findings, ^18^F-FDG PET/CT scans play a remarkable role in predicting histology in thymic disorders. Correlations with findings from the CT component of PET-CT might help in tumor characterization, even though ^18^F-FDG uptake in the thymus can be present in thymic hyperplasia [72,73,74,76]. It has been standardized that the uptake in an SUV value of 3.4 can be a good predictor of malignancy [75]. In difficult cases, chemical shift magnetic resonance imaging (MRI) may be used, with thymic hyperplasia typically demonstrating a decrease in signal on opposed-phase images compared with in-phase images [72,73,74,77,78]. ^18^F-FDG PET/MRI, usually combined with tissue characterization through multiparametric MRI sequences, provides functional and metabolic data from PET and can be used to further improve thymic tissue characterization [79].

As the thymus plays a key role in the defense against viral infections, its importance has been progressively recognized as one of the determining factors in COVID-19 outcomes [38,39,40,41,70,71].

Some authors have speculated that thymic changes play a role in determining the prognosis of COVID-19 patients [38,39,40,41,80]. In fact, thymus enlargement in COVID-19 patients was frequent and associated with increased T lymphocyte production, which appears to be a beneficial adaptation to virus-induced lymphopaenia [38,39,40,41]. The lack of thymic activity/reactivation in older SARS-CoV-2-infected patients could contribute to a worse prognosis [31,32,33,34,35,36,37,38,80]. Different studies found an inverse correlation between pneumonia extent, evaluated by the CT-SS, and the thymus volume and density [38,39,40,41,81]. On the other hand, cases of thymus hyperplasia were found more frequently in COVID-19 patients than in non-COVID-19 cases [41,42].

In some reports, the diagnosis of thymus hyperplasia was incidentally made in COVID-19 patients [38,39]. However, different cases of thymic hyperplasia after COVID-19 vaccination have also been reported [42,43,44,45,46]. In the study of Luthria et al. [42], the authors analyzed the ^18^F-FDG PET/MRI scans of six children with extrathoracic cancer before and after COVID-19 vaccination, and they found an increase in ^18^F-FDG uptake with the restricted diffusion of locoregional lymph nodes and the thymus after receiving COVID-19 vaccination. The observed decrease in the mean ADC values of the thymus after vaccination indicates the increased cellularity of the thymus tissue, which could be related to immune cell infiltration or proliferation in response to the vaccination. Nevertheless, the majority of reported cases regarded small case series or reports with ^18^F-FDG PET/CT scan evaluations, and the majority of these cases involved oncologic patients [42,43,44,45,46]. Additionally, the history of recent vaccination, the combination of metabolically active thymus and lymph nodes, and the absence of bone marrow activation led to the correct diagnosis.

### 3.3. Myasthenic Syndrome After SARS-CoV-2 Infection and Vaccination

The pathogenesis of MG is complex and heterogeneous. Bacterial and viral infections, as well as vaccines, can trigger myasthenic crises and may exacerbate an already existing but asymptomatic form of MG or be associated with new-onset MG, even in previously healthy individuals [47,48,49,50,51,52,53,54,55,56]. Some virus infections—such as the Epstein–Barr virus, hepatitis E virus, West Nile virus, and human parvovirus B19—correlated with the occurrence of MG. The correlation of vaccines and MG syndrome has been previously reported following influenza, human papillomavirus, or hepatitis B vaccinations [43,47,52,53,57].

The exact mechanism by which vaccines or infections could induce autoimmune neuropathies remains unknown. Nevertheless, it can be caused by the molecular mimicry in which the immune response would cross-react with self-antigens if vaccine or viral antigens mimic self-antigens [52,53,57]. On the other hand, genetic susceptibility and an abnormal thymus play an important role in MG pathogenesis [57]. Thymic dysfunction is a well-recognized co-factor of the disease, and MG patients may have a family history of autoimmune illnesses [47,52,57,61]. In fact, the thymus plays an important role in the regulation of the immune response as it is a specialized primary lymphoid organ responsible for the development of mature, self-tolerant T cells and the T cell-mediated immune response [69,70]. However, thymic pathologies are observed in approximately 80% of patients with acetylcholine receptor antibody-positive MG (AChR-MG). Thymic hyperplasia is usually associated with an early-onset MG, and in some patients, it is associated with late-onset MG, ocular MG, and seronegative disease [57]. Thymic hyperplasia is very common in patients with triple-negative MG and was observed in 20.2% of patients [61].

Younger patients were more likely to relapse. However, good outcomes were observed with clinical improvement and remission after surgical resection [57].

Nonetheless, an increasing number of new-onset MG cases have been reported during the COVID-19 pandemic, particularly in patients with longer hospital stays after symptom onset—often following COVID-19 vaccination, especially after the first dose [47,53].

COVID-19 infection, in fact, can potentiate auto-inflammatory mechanisms and work as a trigger for MG symptoms due to structural similarities between the Ach receptor and the SARS-CoV-2 surface receptor [47,52,53]. Moreover, COVID-19 mRNA vaccines could be a trigger in the autoimmunity process, as they contain dsRNA or other analogs that could cause thymus-associated MG [47]. Huang et al. [47] reported that the vaccination rate in patients with new-onset MG after the outbreak of COVID-19 was higher. Most published cases reported that MG manifestations occurred within 1 to 20 days after the vaccine administration, and they were more frequent in the elderly population.

SARS-CoV-2 vaccination has been reported in association with exacerbations of a range of autoimmune and non-autoimmune disorders, including inflammatory arthritis, pericarditis, myocarditis, psoriasis, vasculitis, autoimmune haemolytic anaemia, immune thrombotic thrombocytopenia, Guillain–Barré syndrome (GBS), venous sinus thrombosis (VST), and transverse myelitis [43,47,52,53].

Therefore, both COVID-19 vaccination and infection could also be triggers in an already existing but asymptomatic form of MG in people with genetic predisposition or susceptibility linked to some HLA haplotypes, such as HLA DQA1 and DQB1 [57]. Genes that influence thymic function and T cell maturation also play a role in autoimmune conditions such as myasthenia gravis and pure red cell aplasia, which are frequently associated with thymic disorders [57]. In our cases, even if MG syndrome was not demonstrated, the surgical intervention of thymectomy improved both the mother’s and son’s symptoms. However, the diagnosis of TN-MG was hypothesized in the son based on clinical history, although it was not supported by the findings of electromyography changes. In fact, the boy also responded to pyrostigmina. On the other hand, our cases highlight how familiar and genetic susceptibility can be important in MG-like manifestations, potentially pointing to autoimmunity mechanisms with thymic involvement, as the boy and his mother had similar findings.

### 3.4. Long COVID (PASC) and Long Post-Acute COVID-19 Vaccine Syndrome (LPACVS)

Long COVID (also termed “post-acute sequelae of COVID-19” or PASC) refers to a multisystemic debilitating condition characterized by the persistence of symptoms after COVID-19 infection [36,37,82]. The common symptoms of PASC include fatigue, dyspnea, myalgia, chest pain, coughing, and sputum production, but they can also include symptoms associated with different processes in all organ systems [36,37,82]. Some authors have suggested that gut microbiota imbalances are implicated in the exacerbation or maintenance of diverse long COVID manifestations, particularly those involving fatigue, musculoskeletal pain, gastrointestinal disturbances, and neuropsychiatric symptoms such as depression, anxiety, and headache [82,83,84,85]. On the other hand, it has been speculated that the dysbiosis observed in COVID-19 patients drives inflammation and contributes to long-term symptoms [83,84,85,86].

According to the World Health Organization (WHO) and the US Centers for Disease Control and Prevention (CDC), PASC is characterized by the continuation or development of new symptoms 4 weeks or more after the initial SARS-CoV-2 infection, with these symptoms lasting for at least 2 months with no other explanation [37]. PASC symptoms can last for years and are usually more frequent in hospitalized patients [36,37].

In our case, the boy also presented with a cough as a persistent symptom after the first infection as a long COVID manifestation. However, there is also a recently recognized clinical condition after COVID-19 vaccination with symptoms similar to long COVID, classified as long post-COVID-19 vaccination syndrome (LPCVS) or long post-SARS-CoV-2 vaccination syndrome, which occurs after SARS-CoV-2 vaccination and lasts for >4 weeks [36,62,63,87].

Side effects of SARS-CoV-2 vaccines lasting > 4 weeks include visual disturbances, myalgia, muscle cramps, chronic fatigue and muscle weakness, syncope, fever, headache, palpitations, and dyspnea [46,62,63]. Several authors postulated a common biological pathway between PASC and LPCVS, involving a shared toxin—the spike protein—and elevated levels of pro-inflammatory cytokines [36,62,63,82,87,88]. On the other hand, the spike protein can trigger inflammation across multiple organs and systems [36,62,63,88,89,90]. In addition, both natural and vaccine spike proteins may still be present in patients with long COVID symptoms, thus supporting the existence of a possible mechanism that causes the persistence of spike proteins in the human body [36,62,63,87,88,89,90]. Furthermore, the spike protein has been detected several weeks after COVID-19 vaccination, raising concerns about its potential to induce prolonged or chronic inflammation in various organs [36,88,89,90]. Additionally, the concentration of free spike proteins in circulation after vaccination has been reported to reach levels comparable to those observed during SARS-CoV-2 infection, potentially activating ACE2 receptors, particularly in tissues with high spike protein accumulation [88,89]. Recently, Patterson et al.’s [90] study showed that both infection and vaccination can result in the persistence of the S1 subunit of the SARS-CoV-2 spike in CD16+ monocytes, sustaining inflammation in both PASC and PCVS through vascular pathways with the upregulation of IL-1, IFN-γ, and TNF-α in endothelial cells. Activated platelets recruit monocytes and neutrophils, consistent with symptoms such as fatigue, tremors, and chest pain. Elevated VEGF levels may worsen microvascular permeability and thrombosis. On the other hand, the study of Mantovani et al. [91] reported a higher prevalence of autoantibodies against several antigens involved in the regulation of the autonomic nervous system and the renin–angiotensin system in patients with LPACVS.

Nevertheless, the role of the thymus in LPACVS is not yet fully understood. However, it has been theorized that its involvement may relate to its central role in immune cell development and its association with autoimmune disorders [36,57,61]. The thymus is crucial for the education of T cells to distinguish the body’s own proteins from foreign invaders. Furthermore, this process could be affected and disrupted by exposure to spike proteins, potentially resulting in autoimmune cross-reactions and contributing to post-vaccination immune dysregulation or toxicity. On the other hand, viral infections can also cause epigenetic events that result in various clinical phenotypes. Understanding these complex mechanisms, which can interact or cross-react, may offer valuable therapeutic options for precision medicine control and management [92].

However, more multicentric studies with a control group are necessary to confirm the role of the thymus in the response to vaccinations and infections, as we only describe two representative cases.

## 4. Conclusions

PM in COVID-19 infections can have a multifaceted presentation and a heterogeneous origin. The term spontaneous PM was overused during the pandemic, as few cases were really spontaneous, and most were secondary to COVID-19 pneumonia changes and NIV. SPM, similarly to Hamman’s syndrome, in patients with COVID-19 infection may involve spike protein toxicity and alterations of the lung microbiome. Both COVID-19 infection and vaccination can engage common biological pathways related to spike protein and chemokine toxicity. They may stimulate the thymic response, causing myasthenic-like symptoms as well as long COVID and post-COVID-19 vaccination syndrome. Our two cases—the boy and his mother—demonstrated the importance of genetic predisposition in the thymic response. Interestingly, genes that influence thymic function and T cell maturation play a role in autoimmune conditions, which are frequently associated with thymic disorders and may be implicated in myasthenic-like presentation after infection and vaccination.

## Figures and Tables

**Table 1:** This table reports the laboratory values of the boy in the emergency section in the first column; the second column shows the reference values.

Laboratory Parameters	Value (Units)	Reference Value
**Hemoglobin**	15.00	13.5–17.5
**Neutrophils**	2700/mcL	2000–7000
**Lymphocytes**	1800/mcL	1000–4000
**C-reactive protein**	1 mg/L	<5
**D-Dimer**	0.5 mg/L	<0.5

**Table 2:** This table reports the laboratory values of the mother in the emergency setting in the first column, and in the second column, the reference values are provided.

Laboratory Parameters	Value (Units)	Reference Value
**Hemoglobin**	13.3	13.5–17.5
**Neutrophils**	4000/mcL	2000–7000
**Lymphocytes**	4000/mcL	1000–4000
**C-reactive protein**	0.05 mg/L	<5
**D-Dimer**	1 mg/L	<0.5

**Table 3:** This table summarizes the main case studies and series of pneumomediastinum (PM) in non-mechanically ventilated (MV) COVID-19 patients, reporting the main publications, the timing of the pandemic evaluation, the number (N) of patients evaluated, and the causes of non-MV-PMS. CPAP: Continuous positive airway pressure (CPAP); HFNC: high-flow nasal cannula; NIV: non-invasive ventilation; O2: oxygen therapy; SP: spontaneous pneumothorax; SPM: spontaneous pneumomediastinum.

PMS in Non-MV COVID-19 Patient Case-Studies: First Author and Reference	COVID-19 Wave Period of the Time	N of Patients Evaluated	Causes of Non-MV-PM
Gulati et al. [7]	January 2020 to April 2021	37	23 HFNC and NIV; 11 without NIV 4 without NIV/HFNC
Tetaj et al. [15]	April 2020 to April 2021	2480	221 NIV 4 patients without NIV
Khaire et al. [16]	May 2020 to May 2021	2600 during the 1st wave and 3089 during the 2nd wave	SPM 26 during the 1st wave; 40 during the 2nd wave; 30 NIV 13 HFNC 13 O2
Tekin et al. [18]	April 2020 to January 2022	6637	34 NIV 39 HFNC 33 no high-pressure respiratory support
Marza et al. [19]	March 2020 to October 2022	190	SP-SPM: 15 in wave 1; 32 in wave 2; 46 in wave 3; 29 in wave 4; 68 in the wave 5.
Palumbo et al. [20]	February 2020 to December 2021	221	20 SPM
Tacconi et al. [21]	1st and 2nd epidemic waves	687	7 NIV 2 unassisted breathing
Staiano et al. [22]	January 2020 to November 2020	500	5 HFNC
Haberal et al. [23]	April 2020 to October 2020	38,492	7 SPM O2
Elabaddi et al. [24]	August 2020 to April 2021	549	21 NIV
Palumbo et al. [25]	March 2020 to June January 2021	1151 in the 1st pandemic wave; 1484 in the 2nd pandemic wave	1 SPM in the 1st wave and 13 in the 2nd wave; 8 CPAP in the 2nd wave 6 O2 All patients had corticosteroid therapy
Gandolfo et al. [26]	October 2021 to January 2021	111	11 NIV 6 HFNC

## Data Availability

The original contributions presented in this study are included in the article. Further inquiries can be directed to the corresponding author.
